# Thermal and Mechanical Behavior of New Transparent Thermoplastic Polyurethane Elastomers Derived from Cycloaliphatic Diisocyanate

**DOI:** 10.3390/polym10050537

**Published:** 2018-05-16

**Authors:** Andrzej Puszka

**Affiliations:** Department of Polymer Chemistry, Faculty of Chemistry, Maria Curie-Sklodowska University, ul. Gliniana 33, 20–614 Lublin, Poland; andrzej.puszka@umcs.pl; Tel.: +48-81-524-22-51

**Keywords:** transparent polyurethanes, carboxylate elastomers, thermal and mechanical properties, dynamical mechanical analysis, optical properties

## Abstract

New transparent thermoplastic polyurethane elastomers (TPURs) with the hard-segment content of ≈50 mass % were synthesized by one-step melt polyaddition of 1,1′-methanediylbis(4-isocyanatocyclohexane), 2,2′-methylenebis[(4,1-phenylene)-methylenesulfanediyl]diethanol (diol E), 3-hydroxy-2-(hydroxymethyl)-2-methylpropanoic acid (DMPA), a poly(oxytetramethylene) diol of ${\bar{M}}_{n}$ = 1000 g/mol (PTMO), or a poly(hexametylene carbonate) diol of ${\bar{M}}_{n}$ = 860 g/mol (PHCD). Herein, I prepared TPURs in which 20, 40, and 60 mol % of diol E was replaced with DMPA, an ionic chain extender. The structure of polymers was examined by ATR-FTIR. Their thermal and mechanical behaviors were determined by means of thermogravimetric analysis (TGA), differential scanning calorimetry (DSC), dynamic mechanical thermal analysis (DMTA), tensile tests, and Shore A/D hardness. Their optical properties are described. Generally, the addition of carboxyl groups to the polymer resulted in decreases in their thermal stability, transparency, and refractive indexes. Furthermore, the use of different soft segments revealed significant differences in both mechanical and thermal properties of the polymers obtained.

## 1. Introduction

Polyurethane elastomers are a very interesting type of polyurethane materials. They are now widely used due to their unique properties, such as an outstanding mechanical strength, good chemical resistance, and excellent elasticity. In recent years, the importance of thermoplastic polyurethane elastomers (TPURs) has grown as a result of a less complicated and less expensive production processes compared to vulcanized elastomers, as well as due to the possibility of recycling. TPURs are polymers that show properties characteristic for elastomers in the normal conditions of use getting plasticized when heated. This means that they may be processed using methods typical for thermoplastics, i.e., extrusion, calendering, or injection [1,2,3,4]. Typical TPURs are multi-block copolymers consisting of alternating flexible “soft” segments derived from aliphatic linear polymer diols and”hard” segments formed from diisocyanates and short-chain diols [1,3,4,5,6]. TPURs are polyaddition reaction products involving aromatic (mainly 1,1′-methylenebis (4-isocyanatobenzene) or aliphatic diisocyanates (predominantly 1,1′-methylenebis (4-isocyanatocyclohexane) (HMDI) and 1,6-diisocyanatohexane), aliphatic linear polymer diols (polyether diols, polyester diols or polycarbonate diols), as well as chain extenders (usually butane-1,4-diol), which require the presence of a catalyst [1,2,3,4,5,6,7,8,9] in the case of less reactive substrates.

The soft segments confer the polyurethanes with their softness, elasticity, long elongation at break and low-temperature resistance, whereas the hard segments particularly affect the modulus of elasticity, hardness, and tear strength. Oligoester, oligoether, or oligocarbonate diols are the most used oligomeric diols to synthesize conventional thermoplastic polyurethanes. The polyurethanes based on the latter diols, which are relatively new, have at the same time a high resistance to heat, hydrolysis and oxidation in comparison with polyurethanes derived from oligoether and oligoester diols [10,11,12,13,14]. Soft segments based on polyester, polyether, or polycarbonate oligomers have glass transition temperatures below room temperature and provide TPURs with flexibility, while hard segments produced through the reactions of diisocyanates and chain extenders (such as low molecular weight diols) behave as a reinforcing nano-sized fillers and provide TPURs with tensile strength and rigidity [15,16,17]. This special structure causes its special properties. For example, Young’s modulus, between rubber and plastics, provides TPURs with excellent properties, such as resistance to wear, to solvents, and to oxidation, anti-aging properties, superior toughness, and impact resistance, high tear strength and elongation, etc. Moreover, hardness, flexibility, and elasticity of TPURs can be adjusted over a wide range of raw and processed material [18,19,20]. The thermodynamic incompatibility of soft and hard segments, often combined with crystallization of either or both segments, drives their micro-phase separation into hard and soft phases that are respectively below and above their glass transition temperatures. This micro-phase separation is responsible for the excellent elastomeric properties of these polymers [1,2,3,17].

This paper is a continuation of the research on the new TPURs derived from aliphatic-aromatic sulfur-containing chain extenders as well as derivatives of diphenylmethane [4,5,21,22,23,24,25,26,27]. Based on the test results, it can be concluded that the introduction of sulfur atoms into the structure of polymers increased their adhesive strength [5,24] and refractive index [5,24,25]. In addition, antibacterial properties against Gram-positive bacteria were indicated [5,22].

In this work, it was present both synthesis and characterization of HMDI-based TPURs containing carboxylic groups. These polymers were obtained by modification of previously described TPURs [5] by means of 3-hydroxy-2-(hydroxymethyl)-2-methylpropanoic acid (DMPA). The introduction of functional groups into the polymer structure, sulfonic and carboxylic, makes it possible to improve the biocompatibility of these materials and thereby use them in the production of various medical appliances [28,29]. Additionally, their presence makes it possible to obtain ionomers that can be utilized in the production of, inter alia, coating materials [4,28,29,30,31,32,33,34].

## 2. Materials and Methods 

### 2.1. Materials and Synthesis

The 2,2′-methylenebis[(4,1-phenylene)-methylenesulfanediyl]diethanol (diol E) (m.p. = 77–78 °C) was obtained by a condensation reaction of [methylenedi(4,1-phenylene)]dimethanethiol with 2-chloroethanol in a 10% aqueous solution of sodium hydroxide [24]. PTMO and PHCD were purchased from Sigma-Aldrich (St. Louis, MO, USA). Before being used, PTMO and PHCD were heated at 90 °C in vacuo for 10 h. HMDI (Desmodur W*^®^*) was kindly supplied from Covestro (Leverkusen, Germany). Dibutyltin dilaurate (DBTDL) from Merck-Schuchardt (Hohenbrunn, Germany) and DMPA from Sigma-Aldrich (Steinheim, Germany) were used as received. 

**Scheme 1:**
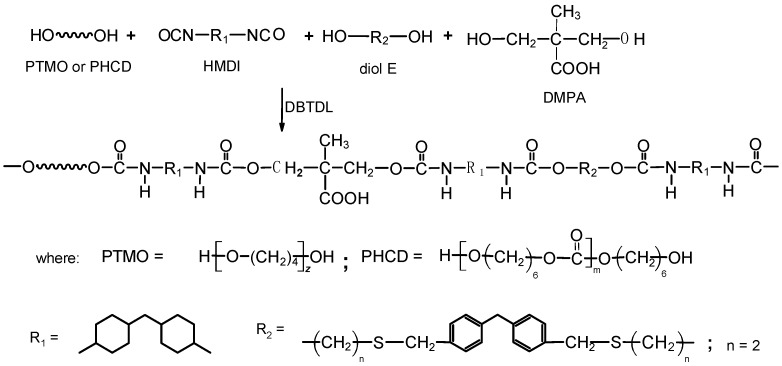
General scheme of synthesis of the thermoplastic polyurethane elastomers (TPURs).

All TPURs were prepared according to Scheme 1, by the one-step melt polyaddition process from HMDI, diol E, DMPA, PTMO, or PHCD at the NCO/OH molar ratio of 1.05.

The general procedure for the synthesis of TPURs by this method was as follows: PTMO or PHCD**,** diol E, and DMPA (0.01 mol together) and HMDI (0.0105 mol) were heated with stirring under dry nitrogen to 95 °C in an oil bath. A catalytic amount of DBTDL (about 0.03 g, 4.52 × 10^−5^ mol) was added to the clear melt formed and polymerization rapidly began at vigorous stirring. The reaction temperature was gradually raised to 130 °C and the colorless rubber-like product formed was additionally heated at this temperature for 2 h. In this way, it was prepared TPURs whose 20%, 40%, and 60% of diol E was replaced with an ionic chain extender DMPA. These polymers were designated as X-D20, X-D40, and X-D60, where X represents the type of soft segments (P: PTMO; C: PHCD) [4,5]. Designations and composition used to synthesize the TPURs are given in Table 1.

### 2.2. Methods

#### 2.2.1. Attenuated Total Reflectance - Fourier Transform Infrared Spectroscopy (ATR-FTIR)

ATR-FTIR spectra were obtained with a Bruker TENSOR 27 FTIR (Ettlingen, Germany) spectrophotometer equipped with Pike Miracle single-bounce attenuated total reflectance (ATR) cell with a diamond/ZnSe crystal plate. Spectra were recorded from 4000 to 600 cm^−1^ averaging 32 scans with a resolution of 4 cm^−^^1^ in the absorbance mode. The TPURs were in the form of the compression-molded 1 mm thick sheets.

#### 2.2.2. Thermogravimetric Analysis (TGA)

Thermogravimetric analysis (TGA) was performed with a Netzsch STA 449 *F1 Jupiter* thermal analyzer (Selb, Germany) in the range 40–700 °C in helium (gas flow = 20 cm^3^/min) and at the heating rate of 10 °C/min. Sample masses of about 10 mg were used. All measurements were taken in Al_2_O_3_ crucibles (with mass about 160 mg) and as a reference empty Al_2_O_3_ crucible was employed.

#### 2.2.3. Differential Scanning Calorimetry (DSC)

DSC thermograms were obtained using Netzsch 204 calorimeter (Günzbung, Germany). The sample of 10.0 ± 0.05 mg was weighed and was first cooled and isotherm for 3 min at −100 °C and then heated up to a maximum temperature of 200 °C, next cooled to −100 °C, and then heated to 200 °C. The scans were performed at the heating/cooling rate of 10 °C/min under nitrogen atmosphere (gas flow = 30 cm^3^/min). All DSC measurements were taken in aluminum pans with a pierced lid (a mass of 40 ± 1 mg). As a reference, an empty aluminum crucible was applied. The reported transitions were taken from first and second heating scans. Glass-transition temperatures (*T*_g_s) for the polymer samples were taken as the inflection point on the curves of the heat-capacity changes. Melting temperatures (*T*_m_s) were read at endothermic-peak maxima.

#### 2.2.4. Optical Properties

● Transmittance

The ultraviolet-visible (UV/vis) spectra of the compression-molded sheets of the TPURs were obtained by a UV-2550 (Shimadzu, Kioto, Japan) UV spectrophotometer in the range of 200–900 nm and at a scanning rate of 200 nm/min.

● Refractive Index

Refractive index was measured at 23 °C by using Conbest Abbe’s Refractometer Type 325 (Krakow, Poland) instrument according to Method A of European Standard EN ISO 489:1999. 1–Bromonaphthalene was applied between the sample film and the prism shield.

#### 2.2.5. Dynamical Mechanical Thermal Analysis (DMTA)

DMTA of TPURs was performed in tensile mode using DMA Q800 Analyzer TA Instruments (New Castle, DE, USA). Calibration was performed as per the manufacturer’s recommendations included in Advantage Software, version 5.5.24 (TA Instruments, New Castle, DE, USA). The experiments were carried out on rectangular samples of dimensions close to 1 mm thick, 5 mm wide, and 30 mm long. Experimental conditions employed were frequency of 1 Hz and static stain 0.05% with the scanning temperature range from −100 °C to 180 °C in the air conditions and a temperature ramp of 3 °C/min. The samples were cut from the pressed sheets. The variations of storage modulus (*E*′), loss modulus (*E*″), and tangent delta (tanδ) versus temperature were determined.

#### 2.2.6. Mechanical Properties 

The readings were taken after 15 s at the temperature of 23 °C. Tensile testing was performed on a Zwick/Roell Z010 tensile-testing machine (Ulm, Germany) according to PN-81/C-89034 at the speed of 100 mm/min at 23 °C; the tensile test pieces 1 mm thick and to Polish Standard 6 mm wide (for the section measured) were cut from the pressed sheet. Press molding was done with a Carver hydraulic press (USA) at 100–120 °C under a 10–30 MPa pressure. The hardness of the TPURs was measured by the Shore A/D method on a Zwick 7206/H04 hardness tester (Ulm, Germany).

## 3. Results and Discussion

The new TPURs were colorless, highly transparent solids. All synthesized polymers were insoluble in DMSO, but easily dissolved in 1,1,2,2-tetrachloroethane (TChE) at room temperature, and some of the polymers were partially soluble, at room or elevated temperature, in *N*-methyl-2-pyrrolidone (NMP), *N*,*N*-dimethylacetamide (DMAc), and DMF (except P-D60, which was fully dissolved in DMF at room temperature). PTMO-based TPURs were also insoluble in THF, but TPURs with polycarbonate soft segments dissolved in this solvent. Generally, with the increase of DMPA content in the polymer, the solubility in these solvents decreased (except for TChE and THF, for which the solubility was the same). As is well known, the degree of solubility TPURs largely depends on their structure, interactions between soft and hard segments, or their molar masses. Generally, linear TPURs are relatively easily soluble in highly polar solvents such as THF or DMF. Their solubility is all the worse, the more rigid segments are ordered and the greater the molar mass of the polymer. In addition, the presence of large amounts of hydrogen bonding solubility also deteriorates [1]. In the case of the tested TPURs, the introduction of polar carboxyl groups resulting in increased cross-linking of the polymers by hydrogen bonding, which involves a reduction in their solubility. Comparing the solubility of TPURs with various soft segments, it can be seen that PTMO-based polymers are more easily dissolved, which may be related to a weaker interaction of hydrogen bonds between soft and hard segments. Moreover, their worse solubility was also probably caused by a higher degree of micro-phase separation (which was confirmed by DSC analysis).

FTIR (cm^−^^1^) of the PTMO-based TPURs: 1725–1722 (nonbonded C=O stretching); 1700–1697 (H-bonded C=O stretching); 3323–3318 (N–H stretching) and 1526–1525 (N–H bending) of the urethane group; 1100–1099 (C–O stretching of the ether group); 1448–1447 (C–H bending of the cyclohexane ring); 2926–2925 and 2854–2852 (asymmetric and symmetric C–H stretching of CH_2_ and CH_3_, respectively); 1369–1368 symmetric C–H bending of the CH_3_ group.

FTIR (cm^−1^) of the PHCD-based TPURs: 1740–1739 (nonbonded C=O stretching of the carbonate group); 1716–1713 (nonbonded C=O stretching of the urethane group and H-bonded C=O stretching of the carbonate group); 1700–1698 (H-bonded C=O stretching) of the urethane group; 1518–1514 (N–H bending) and 3338–3335 (N–H stretching) of the urethane group; 1248–1246 and 969–967 (asymmetric and symmetric C–O stretching of the carbonate group, respectively); 793–790 (C–O bending of the carbonate group); 1454–1451 (C–H bending of the cyclohexane ring); 2927–2926 and 2857–2855 (asymmetric and symmetric C–H stretching of CH_2_ and CH_3_, respectively); 1369–1365 symmetric C–H bending of the CH_3_ group.

**Figure 1:**
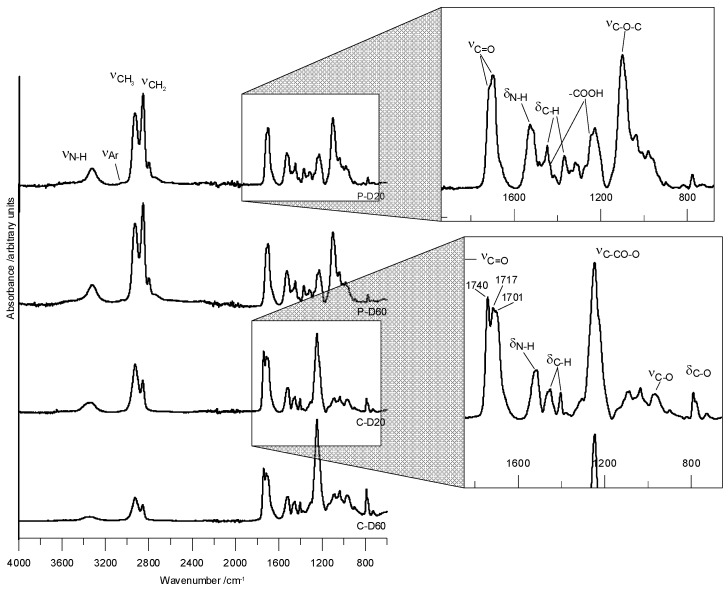
ATR-FTIR spectra of the selected TPURs.

The carboxyl group was identified in peaks in the range of about 1430 and 1255 cm^−1^, which were clearly visible for PTMO derivatives. The carboxyl groups also produce a strong band of carbonyl stretching tensions, which at low concentrations is invisible. Figure 1 presents ATR-FTIR spectra of selected TPURs.

### 3.1. TGA

The thermal stability of TPURs was obtained in inert (helium) atmosphere using TGA analysis. Based on the course of TGA curves, the temperatures of mass loss: *T*_1_, *T*_5_, *T*_10_, and *T*_50_ were designated, while *T*_max_ was determined on the basis of a derivative TGA (DTG) curves. Table 2 presents all the determined values. 

The TPURs obtained exhibit good thermal stability (their temperatures of a 1% mass loss were in the range from 234 to 290 °C), wherein the TPURs with polyether soft segments show a higher *T*_1_ than the corresponding PHCD-based ones. It is evident from a better thermal stability of PTMO than PHCD.

The values of all the TPURs decomposition temperatures (*T*_1_, *T*_5_, *T*_10_, and *T*_50_) in all series decreased with increasing of carboxylic groups in polymer. On this basis, we can state that DMPA addition resulted in the decrease in thermal stability of the polymers obtained. This is due to the fact that the carboxylic group introduced into the polymer chain easily breaks down under high temperature. Additionally, tertiary carbon atom connected to carboxylic group is more prone to be attacked by radicals formed during the decomposition of the polymer [4,33,34]. This interpretation is consistent with the results obtained. 

**Figure 2:**
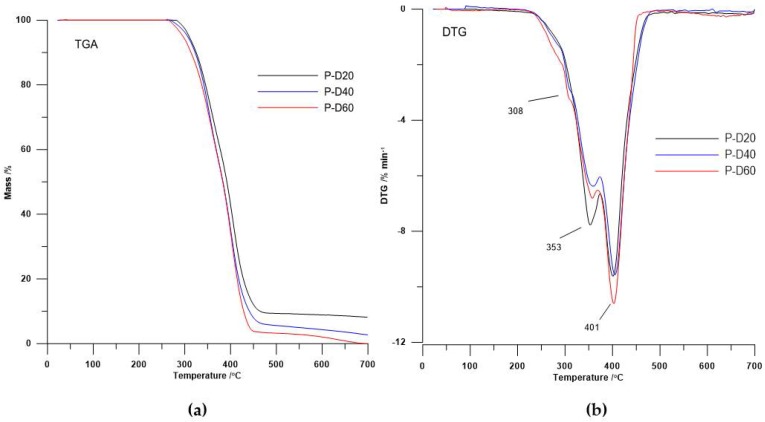
(**a**) TGA curves and (**b**) DTG curves of the PTMO-based TPURs.

**Figure 3:**
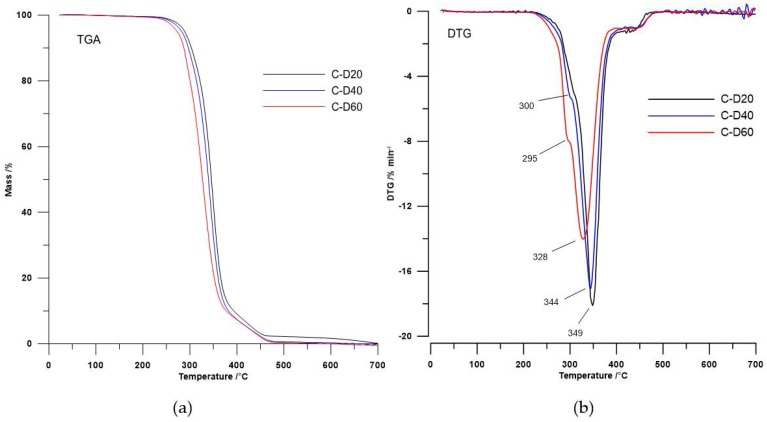
(**a**) TGA curves and (**b**) DTG curves of the PHCD-based TPURs.

The DTG curves course (see Figure 2 and Figure 3) showed that the decomposition of the polymers is a multi-stage process that depends on their physical and chemical structure. Taking account the shape of DTG curves of polyurethanes without DMPA as the chain extender [5] and the shape of DTG curves obtained TPURs, it can, with a high probability, state that DTG peak appears like a small broad peak as shoulder with the maximum temperature at about 308 °C are associated with the decomposition of a hard segments prepared from DMPA and HMDI. In both series of TPURs intensity of these peaks increasing with increasing DMPA content in polymer. The second DTG peak for PTMO-based TPURs (peak with a maximum temperature in the range of 353–360 °C) was associated with the decomposition of urethane and sulfide linkages, whereas the third DTG peak for these polymers (clear, sharp peak with a maximum temperature in the range of 401–420 °C) was connected to the decomposition of ether linkages. 

In the case of polymers based on PHCD, the second DTG peak (a wide peak with the maximum temperature in the range of 328–349 °C) related with the decomposition of urethane, sulfide, and carbonate linkages, while the third DTG peak (a small peak with the maximum in the range of 425–433 °C) was coupled with the decomposition of aromatic content [5,22,23,24].

### 3.2. DSC

**Figure 4:**
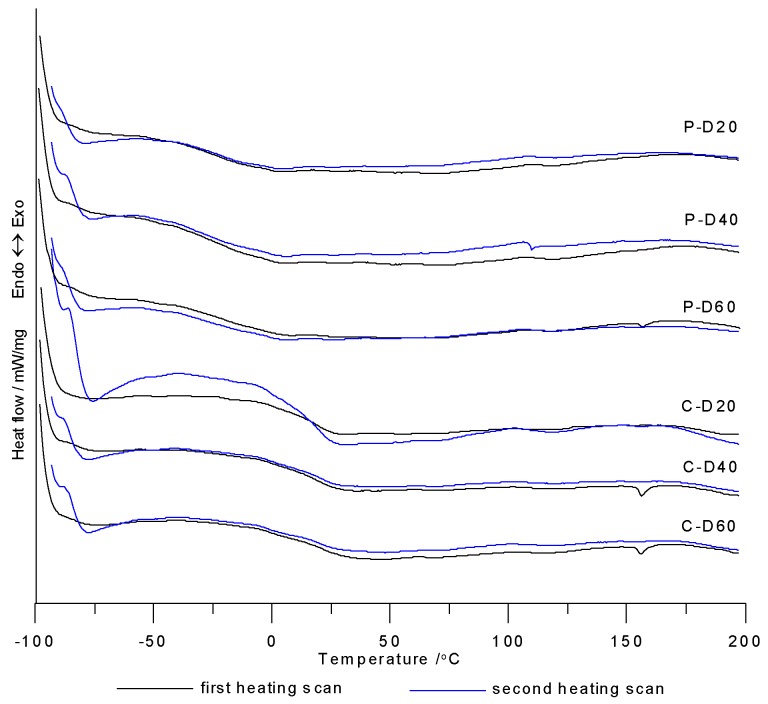
DSC curves of TPURs.

DSC measurements were taken in the course of two heating cycles at temperatures ranging from −100 to 200 **°**C. Table 2 contains numeric data determined from DSC curves from the first and second heating cycles. Figure 4 shows the DSC curves of the synthesized polymers. Glass transition temperatures were visible on the DSC curves of all polymers, where some of them also exhibit small visible endothermic peaks with very low Δ*H* values (from 0.20 to 0.54 J/g). Based on these results, it was concluded that the synthesized polymers were amorphous.

The *T*_g_ values for PTMO-based TPURs ranged from −31 °C to −17 °C, and for PHCD-based ones between 17 and 21 °C. Analyzing the changes in the content of COOH groups in the polymer, it can be seen that, for both series of polymers, this change has no significant effect on the *T*_g_ values (except for the polymer P-D40 for which the *T*_g_ value was the lowest). By comparing the influence of the soft segment types used to synthesize TPURs on *T_g_* values, it can be clearly stated that PTMO-based polymers exhibited significantly better micro-phase separation in comparison to PHCD-based ones. It follows from the differences in *T*_g_ values of the resulting polymer and the corresponding pure soft segment (PTMO-1000: −77 °C, PHCD: −68 °C) [5,10]. The higher degree of micro-phase separation is the result of a weaker interaction of hard-segment urethane groups with ether oxygen compared with carbonate carbonyl groups of soft-segment chains. To some degree, the negligible difference in molar masses of soft segments may also influence the phenomenon mentioned above. The data shown in Table 2 confirm that PHCD-based TPURs revealed a *T*_g_ value at approx. room temperature, which means that they were on the borderline of plastomers and elastomers [5,35]. 

### 3.3. Optical Properties

**Figure 5:**
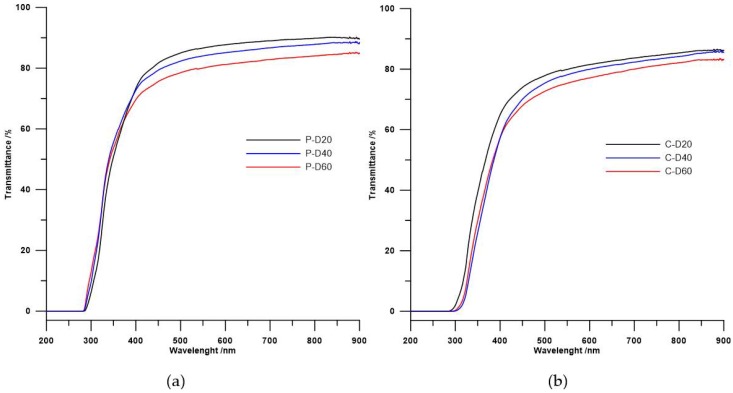
Ultraviolet–visible spectra of the (**a**) PTMO and (**b**) PHCD-based TPURs.

Table 3 presents refractive indexes and transmittance data obtained for all synthesized polymers, and Figure 5 provides the UV/vis spectra of those TPURs. 

UV/vis spectroscopy is limited to polymers with specific chromophores such as aromatic groups, conjugated multiple bonds, or atoms absorbing UV such as S. The data indicate that, in both series of polymers, refractive indexes as well as transmittance decrease with the increase in carboxylic groups (i.e., reduction in sulfur atoms content). The refractive index values largely depend on the content of sulfur atoms in the polymer; hence, the above assumptions and obtained results are consistent with the literature [4,36,37]. 

In case of transmittance, its value may be determined by other factors. According to literature, transmittance depends on inter alia the cross-linking of the probes [4,27] or on the mixing of the soft and hard phase in the polymer [4,5,38]. In both series TPURs, transparency changed in the same directions as the refractive index. The same dependence was seen for the above-mentioned poly(thiourethane-urethane)s [25], whereby the PTMO-based TPURs showed higher transparency than analogous TPURs with a polycarbonate soft segments. These results are inconsistent with theoretical assumptions, so transparency also depends on other factors not previously described. For both series of TPURs received, the decrease in transparency along with the increase in carboxylic groups may also be due to the presence of small ordering, as demonstrated by the DSC analysis (small endothermic peaks at ~156 °C).

### 3.4. DMTA

**Figure 6:**
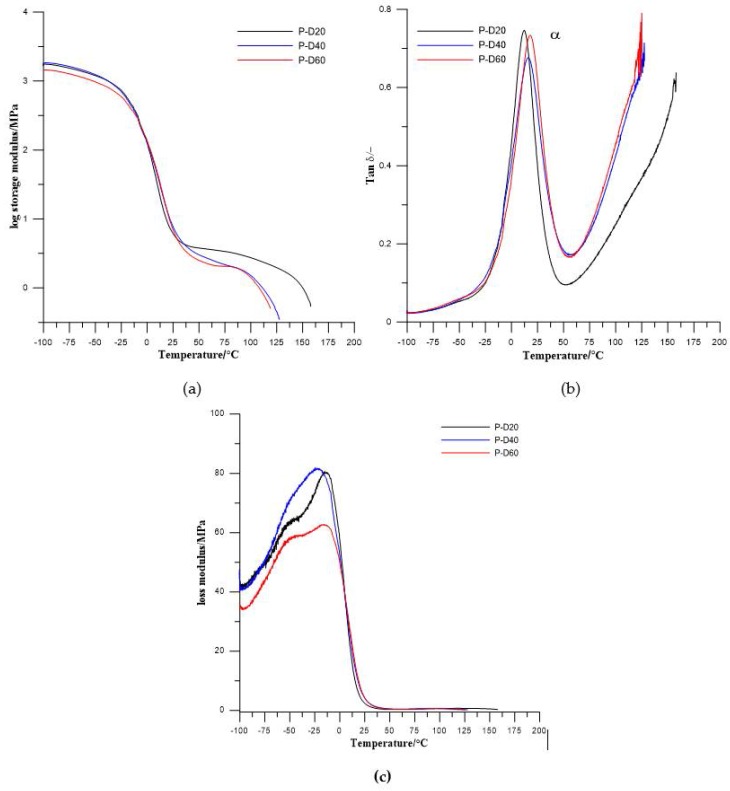
Temperature dependence of (**a**) storage modulus, (**b**) tan delta, and (**c**) loss modulus of the PTMO-based TPURs.

**Figure 7:**
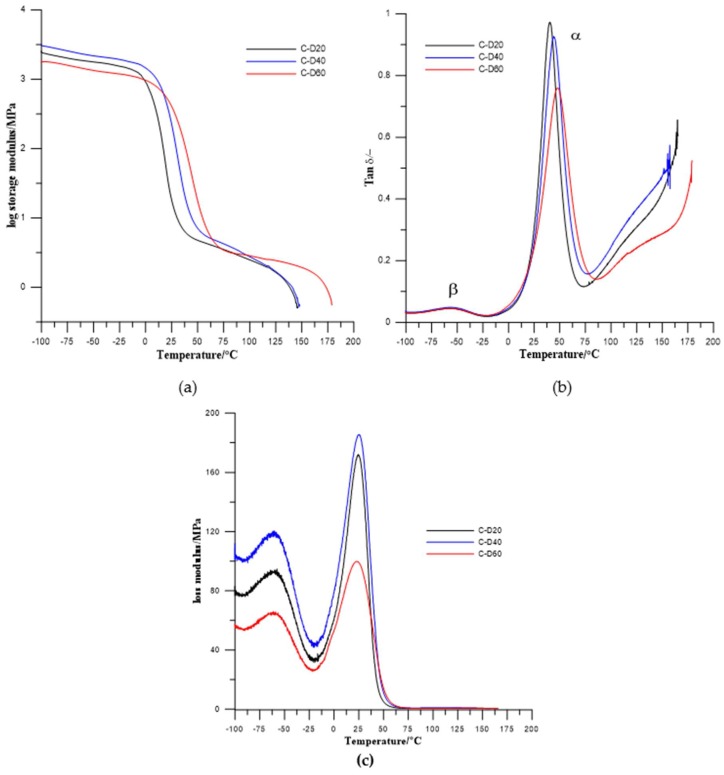
Temperature dependence of (**a**) storage modulus, (**b**) tan delta, and (**c**) loss modulus of the PHCD-based TPURs.

Results from dynamical mechanical thermal analysis of synthesized TPURs are presented in Figure 6 and Figure 7 and Table 4. 

In the obtained results, one (for PTMO-based TPURs) or two (for PHCD-based ones) types of relaxations are visible. First one, coded as α relaxation (with the maximum in the range from 12.1 to 17.6 °C for PTMO-based TPURs and 40.4 to 47.8 °C for PHCD-based TPURs) is primary relaxation connected with the glass transition of the soft segments in the polymer, while the second one is a auxiliary relaxation coded as a β relaxation (with the maximum in the range from −53.1 to −56.5 °C). The β relaxations are evidently visible only for TPURs prepared from PHCD and can be related to the molecular motions in the soft segment main chains, and local motions of the polar urethane groups. From the obtained results, it can be seen that the storage modulus (*E*′) gradually decreases above −20 °C for TPURs with PTMO, and above 12 °C for PHCD-based ones. These changes are related to the onset of the loss of elastic energy state due to heating. The decreasing of *E*′ has a maximum rate at the region connected with the glass transition temperature of soft segments. It is assumed that, in this range, there is a transition from the elastic energy range to the elastic entropy application, and the latter can also be called the softening area of the polymer. In this area, the segments of the chains, in contrast to the elastic energy range, are subject to increased displacements or rotational movements.

After the glass transition, the storage modulus no longer decreases strongly, and, on the curves plateaus are observed. In these ranges, the polymers are in an elastic entropy application state. In these conditions, the ability to rotate and move the segments of the chain allows molecules to accept upright shape under load. The appearance of such upright shapes is thermodynamically unlikely, so chain segments tend to adopt the form of a tangle (a state of increased entropy). Because of this, elastic recovery force is generated. In this area, polymeric materials have rubber-like properties and this state is referred to as rubber-elastic.

Above the entropic and rubber elastic area, with a further increase in the temperature, a significant decrease of *E*′ was observed. In this range, the melting area begins, constituting the transition to the flow range in which the material is in the form of a melt. Due to the mobility of the macromolecules, their entanglement and ordering are easily limited and move away from each other; due to that, the polymers go into the state of an amorphous alloy. In this condition, TPURs (as well as other polymers) can easily be formed using conventional processing processes (blow molding, film and fiber production, etc.).

The glass transition temperatures, estimated as a maximum value of tanδ, were higher for TPURs with polycarbonate soft segments than analogous PTMO-based ones, which is in good agreement with DSC results. The maximum values of tanδ peaks were in the range of 0.55–0.66 for PTMO-based TPURs and 0.66–0.89 for PHCD-based ones. These values indicate that TPURs with PTMO as a soft segment have better vibration damping (absorption) properties. As is known, as the material becomes better able to absorb the vibration, the value of tan*δ* is reduced [39]. In addition, these values show that PTMO-based TPURs have more flexible properties than TPURs with PHCD, as confirmed by the DSC analysis.

### 3.5. Mechanical Properties

The mechanical and thermal properties of the polyurethanes are determined by a number of factors including the molecular weight and type of soft and hard segment, interactions between the hard and a soft phases and crystallinity. It is preferable to describe the relationships between the chemical structure of the polyurethanes and the resultant physical properties.

As can be seen from the data presented in Table 5, the PHCD-based TPURs showed a much higher hardness (72/31–80/36 vs. 53/17–60/24 °Sh in Scale A/D), tensile strength (38.2–41.1 MPa·vs. 4.24–10.1 MPa), a modulus of elasticity (19.1–46.3 vs. 0.09–1.93 MPa), and lower elongation at break (250–300% vs. 425–750%) than the analogous TPURs with PTMO. In the case of PTMO-based TPURs elongation at break increased with the increase in DMPA content in TPURs, while tensile strength and modulus of elasticity decreased. 

**Figure 8:**
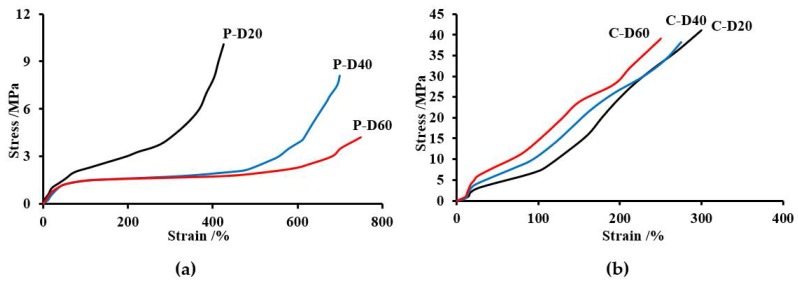
Stress–strain curves of the TPURs obtained from PTMO (**a**) and PHCD (**b**).

In the case of TPURs obtained from the PHCD, the dependence of the modulus of elasticity and the elongation at break on the content of DMPA in TPURs was the reverse of that for polymers from PTMO. This can be explained by stronger intermolecular interaction (including segment polarity, length and stronger hydrogen bonding with the hard segments) in polycarbonate soft segments in comparison with those in polyether ones. In the case of TPURs with a polyether flexible segment, the course of the curves largely depends on the amount of DMPA content in the polymer. The P-D40 and P-D60 polymers exhibit in the elongation range of about 150% to about 400%, a plateau region, after which there is a rapid increase in strength due to the crystallization of the polymer under tension. For those TPURs, the increase in tensile stress shown in Figure 8 had a characteristic course for elastomers (no visible yield stress), which was further confirmed by a small elastic modulus, lower *T*_g_ values obtained by DSC (*T*_g_ values less than the tensile strength measurement temperature), and a high degree of micro-phase separation. In the case of other polymers, the plateau region is invisible. For these TPURs, the curves shown in Figure 8 had a characteristic course for polymers with high hardness and a higher modulus of elasticity compared to those of PTMO. This shape is associated inter alia with a small degree of micro-phase separation, typical for TPURs with polycarbonate soft segments.

## 4. Conclusions

The polymers obtained were colorless, highly transparent materials (up to 89.9%) with elastomeric properties and were characterized by a relatively good thermal stability. Polymers with 5% mass loss were kept within the range of 282–310 °C, while higher values were shown by those based on PTMO as a soft segment. Unfortunately, the addition of DMPA, as an ionic chain extender to the polymers, resulted in the decrease of their thermal stability. DSC analyses showed that all polymers synthesized were amorphous. Polymers with PTMO as the soft segment had significantly lower glass transition temperatures (from –31 to –19 °C) compared with the corresponding ones based on PHCD (from 20 to 21 °C). A comparison of the *T*_g_ of pure soft segments with the polymers obtained evidenced a much greater micro-phase separation ability of the polymers derived from PTMO. The results of the DMTA study of the TPURs showed that favorable elastomeric properties and thus higher damping values are exhibited for PTMO-derived polymers. The hardness of the obtained polymers (in Scale A) ranged from 53 to 80 °Sh, whereas greater values corresponded to the PHCD-based polymers. The TPURs bearing polycarbonate groups as soft segments, compared with the PTMO-based ones, possessed higher tensile strengths (38.2–41.1 vs. 4.24–10.1 MPa) and lower elongation at break (250–300 vs. 320–700%).

## Figures and Tables

**Table 1:** Designations and composition used to synthesize the TPURs.

TPUR	Soft Segment	Amount of HMDI/mol	Amount of Diol E/mol	Amount of DMPA/mol	Amount of Soft Segment/mol	^1^ Hard-Segment content/mass %
P-D20	PTMO	0.0105	0.004	0.001	0.005	46.14
P-D40		0.0105	0.003	0.002	0.005	44.86
P-D60		0.0105	0.002	0.003	0.005	43.53
C-D20	PHCD	0.0105	0.004	0.001	0.005	49.90
C-D40		0.0105	0.003	0.002	0.005	48.62
C-D60		0.0105	0.002	0.003	0.005	47.27

^1^ The hard-segment content (mass %) is calculated by using the expression $\frac{W_{\mathrm{diol}\;E}+W_{\mathrm{DMPA}\;}+W_{\mathrm{HMDI}}}{W_{d\mathrm{iol}\;E}+W_{\mathrm{DMPA}\;}+W_{\mathrm{HMDI}}+\;W_{\mathrm{SS}}}$, where *W*_diol E_, *W*_DMPA*,*_ *W*_HMDI_ and *W*_SS_ are mass of 2,2′-methylenebis [(4,1-phenylene)-methylenesulfanediyl]diethanol (diol E), 3-hydroxy-2-(hydroxymethyl)-2-methylpropanoic acid (DMPA), 1,1′-methylenebis (4-isocyanatocyclohexane) (HMDI) and soft segment, respectively. PTMO: a poly(oxytetramethylene) diol of ${\bar{M}}_{n}$ = 1000 g/mol; PHCD: a poly(hexametylene carbonate) diol of ${\bar{M}}_{n}$ = 860 g/mol.

**Table 2:** TGA and DSC data of the TPURs.

TPUR	*T*_1_^1^/°C	*T*_5_^2^/°C	*T*_10_^3^/°C	*T*_50_^4^/°C	*T*_max_^5^/°C	*T*_g_/°C		*T*_m_/°C		Δ*H*/J/g	
						I ^6^	II ^6^	I ^6^	II ^6^	I ^6^	II ^6^
P-D20	290	309	325	392	353; 401	−21	−19	−	−	−	−
P-D40	279	306	323	383	308;360; 405	−25	−31	−	−	−	−
P-D60	273	296	313	380	308; 358; 403	−17	−21	156	−	0.20	−
C-D20	250	289	303	346	308; 349; 425	20	21	−	−	−	−
C-D40	243	283	297	340	300; 344; 438	17	21	156	−	0.54	−
C-D60	234	273	287	327	295; 328; 433	20	20	156	−	0.45	−

^1,2,3,4^ The temperature of 1%, 5%, 10%, and 50% mass loss from the TGA curve, respectively. ^5^ The temperature of the maximum rate of mass loss from the derivative TGA (DTG) curve; ^6^ I and II—first and second heating scans, respectively.

**Table 3:** Optical properties of the TPURs.

TPUR	Refractive Index	Transmittance/%	
		*T*_500_^1^	*T*_800_^2^
P-D20	1.510	85.0	89.9
P-D40	1.504	82.3	87.8
P-D60	1.501	78.5	84.0
C-D20	1.520	77.9	85.4
C-D40	1.514	75.4	84.2
C-D60	1.507	72.7	82.1

^1,2^ transmittance at 500 and 800 nm, respectively.

**Table 4:** Dynamic mechanical thermal analysis (DMTA) results of the TPURs.

TPUR	^1^ *E*′_onset_/°C	^2^ *E*″_max_/°C	^3^ tanδ*_max_/*°C	tanδ_max_^3^
P-D20	−12.7	−15.1	12.1	0.66
P-D40	−17.7	−17.5	15.6	0.55
P-D60	−13.7	−16.0	17.6	0.61
C-D20	23.5	−57.7; 24.6	−53.1; 40.4	0.89
C-D40	25.8	−59.7; 25.4	−54.7; 43.7	0.82
C-D60	24.4	−61.2; 23.2	−56.5; 47.8	0.66

^1^ storage modulus; ^2^ loss modulus; ^3^ tangent delta (α relaxation).

**Table 5:** Mechanical properties of the TPURs.

TPUR	Hardness/Shore A/D	Tensile Strength/MPa	Elongation at Break/%	Modulus of Elasticity/MPa
P-D20	60/24	10.1 ± 0.24	425 ± 25	1.93 ± 0.02
P-D40	53/17	8.12 ± 0.27	700 ± 14	0.15 ± 0.01
P-D60	53/17	4.24 ± 0.13	750 ± 25	0.09 ± 0.01
C-D20	72/31	41.1 ± 0.27	300 ± 25	19.1 ± 0.04
C-D40	77/35	38.2 ± 0.23	270 ± 14	34.8 ± 0.02
C-D60	80/36	39.0 ± 0.35	250 ± 14	46.3 ± 0.09
